# Ways of knowing the health of livestock populations: the age of surveys, 1928–65

**DOI:** 10.1017/mdh.2023.20

**Published:** 2023-07

**Authors:** Abigail Woods

**Affiliations:** University of Lincoln, United Kingdom

**Keywords:** Veterinary, agriculture, dairy cows, disease survey, twentieth century, Britain

## Abstract

This article advances historical understandings of health, veterinary medicine and livestock agriculture by examining how, in mid-twentieth-century Britain, the diseases of livestock were made collectively knowable. During this period, the state extended its gaze beyond a few, highly impactful notifiable diseases to a host of other threats to livestock health. The prime mechanism through which this was achieved was the disease survey. Paralleling wider developments in survey practices, it grew from small interwar beginnings into a hugely expensive, wide-ranging state veterinary project that created a new conception of the nation’s livestock as a geographical aggregation of animals in varying states of health. This article traces the disease survey’s entanglements with dairy cows, farming practices, veterinary professional politics and government agendas. It shows that far from a neutral reflection of reality, surveys both represented and perpetuated specific versions of dairy cow health, varieties of farming practice and visions of the veterinary professional role. At first, their findings proved influential, but over time they found it harder to discipline their increasingly complex human, animal and disease subjects, resulting in unconvincing representations of reality that led ultimately to their marginalization.

## Introduction

In 1955, delegates to the annual congress of the British Veterinary Association assembled at Queens University, Belfast, to hear a panel of speakers describing their attempts to make the diseases of livestock collectively knowable. Dairy cows were under investigation by F.W. Withers of the government’s Central Veterinary Laboratory (CVL) and F.B. Leech of the Rothamsted Agricultural Experimental Station. Working in various parts of the country, they were attempting to survey the causes of disease and ‘wastage’ (defined as the premature disposal of an animal from the herd.)^1^ In Cumbria, G.B.S. Heath of the Penrith Veterinary Investigation Centre was turning his attention to sheep mortality, using farm records and post-mortem inspections to understand the nature and extent of the problem.^2^ Meanwhile, J.F. Gracey, of the government’s Veterinary Research Division in Northern Ireland, measured losses in pigs as part of a wider survey of livestock disease.^3^

In the discussion that followed, speakers and delegates reflected on the practicalities and purposes of surveying livestock disease. For Withers, it was essential to collect ‘as simply and economically as possible data of this kind so that we can get regular and reliable information on the relative importance of different diseases and the losses they cause’.^4^ For A.W. Stableforth, Director of the Ministry of Agriculture’s CVL, such ‘vital statistics’ were important ‘for when one wants to go to Government to show it is necessary to do something to control disease’. They also helped to demonstrate ‘where funds for research should be put’ and ‘what has been done by a certain drug or vaccine’.^5^ However, gathering disease information was not straightforward. Contributors highlighted farmers’ unreliability as observers and informants and the difficulty of enrolling a representative sample of flocks and herds, as demanded by statistical theory. While punched card technology was proving useful in organising and analysing survey data, it remained challenging to capture data on diseases that did not result in death or disposal.^6^

The importance that these veterinary surgeons awarded to disease surveying suggests that the history of this technique may help answer the perennial medical historical question of how private health became public knowledge. Historians of human health have already addressed this question through studies of collected patient histories,^7^ mass hospital post-mortems,^8^ cause of death statistics,^9^ reports of notifiable diseases^10^ and surveys of health and nutrition,^11^ which together reveal the development of statistical ways of knowing disease.^12^ However, except for notification, which was applied only to a few animal diseases, historians have neglected to consider the methods used to capture the health of livestock populations.^13^ It cannot be assumed that these methods were the same as in human medicine, because as a younger, smaller and lower status profession, vets’ record-keeping practices, scientific resources and legal obligations were much less developed. The demographics of livestock disease were distinct from human diseases, and could be studied in quite different ways and for different reasons. Consequently, veterinary approaches to them require dedicated attention. Surveys – which relied overwhelmingly on self-reporting of disease by farmers – were particularly important, as shown by the more than one hundred articles published on cattle disease surveys in Britain between 1931 and 1961. Another twenty-nine were published on sheep; forty-five on birds, laboratory animals, mink and rabbits; and eleven on multiple species. A further eighty-five survey publications appeared between 1961 and 1965.^14^

Drawing on these outputs, and on a wealth of government archival materials, this article traces the history of livestock disease surveying in Britain. It focusses on the mid-twentieth century, when surveys became the key method of ‘seeing’ the ‘diseases of everyday occurrence, which in the aggregate cause large losses in money and stock’.^15^ Traditionally, such ‘everyday’ diseases had fallen outwith the state’s remit, which extended only to a handful of diseases that it judged particularly damaging to human health or the agricultural economy. However, for reasons that I will explain, in the period under investigation, everyday diseases ceased to be regarded as purely private farming problems and became matters of public concern. This shift was both reflected in and driven by disease surveys. Against a backdrop of developments in veterinary science and practice, and agricultural and public health policy, I trace their initiation by interwar agricultural scientists, extension by wartime veterinary lobbyists and post-war entry into mainstream state veterinary activity. The story culminates in the first ever national surveys of dairy cow and calf diseases, circa 1958–63, and ends by explaining why the method fell out of favour. Applications to dairy cows – as the most economically important and closely scrutinised of all livestock species – are foregrounded throughout.^16^

Surveys were not, of course, unique to livestock health. They developed also in agriculture,^17^ social science^18^ and medicine.^19^ Some scholars have argued that like other statistical ways of knowing populations, surveys formed part of the rationalising apparatus that enabled social and political regimes to discipline and govern their populations at a distance.^20^ Others favour more empirically based interpretations that draw attention to the social, political and economic factors that influenced not only the conduct but also the impacts and reception of statistical ways of knowing.^21^ This article draws on these observations, particularly on John Law’s reflection that as a form of knowledge-practice, the sustainability of surveys depends on their ability to accomplish two tasks simultaneously:

to be able to create knowledge (theories, data, whatever) that *work*, that somehow or other hold together, that are convincing and (crucial this) do whatever job is set for them. But then secondly and counterintuitively, they have to be able to *generate realities* that are fit for that knowledge.^22^

As I will show, surveys produced rather selective knowledge about dairy cow diseases. They were designed to make visible a subset of udder and reproductive diseases that a particular type of dairy farmer found especially problematic. They also extended and perpetuated this partial version of the pathological bovine body by stimulating scientific investigation of these diseases and encouraging vets to orient their science and practice around them. For a time, livestock disease surveys proved highly influential. However, as they developed in scale and statistical sophistication, surveys found it increasingly difficult to discipline their increasingly complex human, animal and disease subjects. Their representations of reality became less and less convincing, leading eventually to their displacement from the centre to the margins of state veterinary medicine.

## Constructing the livestock disease survey, 1928-45

During the late 1920s and 30s, agricultural researchers from the National Institute for Research in Dairying, Reading, the Institute of Animal Nutrition at the School of Agriculture, University of Cambridge and the Hannah Institute in Ayrshire, Scotland, conducted the first systematic surveys of the causes of ‘wastage’ in dairy cows. These surveys broke new ground in their scale (enrolling 320, 550 and 450 herds, respectively), their focus on dairy cows and their use of statistics.^23^ Although there was a tradition of agricultural societies seeking to build information about livestock diseases by circulating questionnaires to their members,^24^ there were few other mechanisms for capturing the health of livestock populations. The state’s role was restricted to a handful of notifiable diseases like foot and mouth disease, anthrax and, increasingly, bovine tuberculosis (bTB).^25^ In contrast to the situation in many continental countries, there was only piecemeal veterinary inspection of livestock carcasses in slaughterhouses.^26^ Few farmers and practising vets kept records of their cases, and the diagnostic support provided to practising vets by state-funded veterinary laboratories was still in its infancy.^27^

Building on domestic and colonial applications during the nineteenth and early twentieth centuries, the survey technique was then gaining credibility as a method of making populations knowable and manageable. This was due to new statistical methods and new fields of application that reflected the state’s expanding responsibilities. Surveys were used to study, objectify and quantify ‘problematic’ human populations, such as the poor, the ill-nourished and the unemployed. In Africa, the Carnegie Foundation sponsored an extensive and highly influential survey of how science was being applied to environmental, medical, racial and anthropological problems.^28^ Surveys of agricultural practices were also increasing, resulting in the 1936 launch of an annual Farm Management Survey that continues to this day.^29^

The wastage surveys conducted at Reading, Cambridge and the Hannah were precipitated by a developing body of research on the physiology of milk production. Using statistical methods pioneered by men connected to the eugenics movement (notably Francis Galton, Karl Pearson and Ronald Fisher),^30^ agricultural scientists had demonstrated that the quantity and quality of milk produced by a cow varied throughout life.^31^ Output peaked at seven or eight years and did not greatly decline until twelve years of age. However, in the herds studied, few cows lived that long. Around one-quarter were replaced each year, at significant cost to the farmer.^32^ This discovery raised questions that the wastage surveys were designed to answer about the reasons for the premature disposal of cows from herds.^33^ Survey findings confirmed that the average productive life of a dairy cow was just four and a half years. While there were some regional differences, surveyors attributed around 60% of wastage to disease. Of the other 40%, around half was due to ‘low yield’, which they acknowledged could itself result from disease. They categorised the diseases responsible into ‘reproductive disease’ (sterility and abortion), ‘udder disease’ (mastitis), ‘tuberculosis, Johne’s disease and wasting’ (Johne’s was a wasting condition that resembled bTB), a positive tuberculin test (which diagnosed bTB) and ‘death and miscellaneous diseases’.^34^

These findings were not neutral reflections of reality. From the medicines that farmers purchased and the letters they sent to farming journals, it is clear that dairy cows were affected by a wide variety of health problems, such as gastrointestinal complaints, colds and respiratory ailments, injuries and skin problems, birthing troubles, swollen udders and cows that ‘slipped’ (aborted) their calves.^35^ Any of these problems could have an impact on milk yields to the extent that farmers felt it uneconomic to retain the affected cow in the herd. However, wastage surveys were designed to pay particular attention to a subset of diseases that had their seats in the organs of milk and calf production. This focus resonated with national concerns about the agricultural economy and the public’s health, and privileged the disease priorities of a particular group of dairy farmers.

As the interwar agricultural depression deepened, farmers turned increasingly to dairy farming because liquid milk was relatively protected from foreign competition. To support their endeavours, the government funded an expansion of agricultural research. Beneficiaries included the institutes where wastage surveys and the physiological investigations that precipitated them were conducted.^36^ Another important interwar concern was the public’s health and the influence that milk exerted over it. Vitamin research and surveys of child poverty and nutrition were revealing the importance of milk as a nutritious and health-giving food for children, as well as the dangers it posed as a vehicle for diseases like bTB, brucellosis and scarlet fever (in which bovine mastitis was implicated).^37^ Therefore for reasons of public health and agricultural profitability, surveyors focussed their attention on these and other diseases of the bovine udder and reproductive system. In so doing, they privileged the disease experiences of a subset of dairy farmers, for whom wastage was a particular concern.

The concept of wastage had been employed in First World War analyses of military manpower as meaning ‘loss or wastefulness’. It was subsequently extended to labour and resources (including soil and crops).^38^ Its application to dairy farming therefore mobilised an industrial concept of efficiency, in which cows were workers. This usage owed much to the epidemiologist, Major Greenwood, who had incorporated vital statistics into discussions of wastage:

if we use the term vital statistics in a specialised way as merely denoting the relations between entrance to and exit from an industry, so that our industrial ‘death’ rate would then merely be the rate at which entrants to a trade pass out of it… in this sense, ‘death’ or wastage rates for different factories will be prima facie measures of the efficiencies of the respective factories, just as local death rates are of sanitary efficiency.^39^

Wastage was therefore a negative concept. Herds with high wastage rates were framed as economically inefficient because their cows did not live long enough to realise their full potential for high milk yields and quality calves. However, this definition was skewed towards closed herds, in which farmers bred all of their own replacements and expected to retain them throughout their productive lives. By contrast, many interwar dairy farmers kept so-called ‘flying herds’ in which ‘wastage’ was a standard management practice. Instead of breeding their own cows, they purchased them soon after calving and sold them towards the end of the lactation when their milk output naturally declined. The scientists who conducted wastage surveys did not attempt to enrol these types of herds – which were frequently looked down upon by veterinary and agricultural commentators.^40^ Instead, they relied on the 5% of the nation’s cows that were enrolled in Milk Recording Societies.

Organised by agricultural colleges and County Councils under the umbrella of a national scheme devised by the Ministry of Agriculture, Milk Recording Societies introduced a high degree of measurability and surveillance into dairy farming. They attracted a small minority of production-oriented farmers who pursued high milk yields through progressive methods of feeding, breeding, healthcare and general management. It was entirely in line with these farmers’ rationalising, improving agendas to regard wastage as a problem. They monitored cow performance by recording individual daily milk yields, and the dates on which cows gave birth and were sent to the bull. These records made visible the effects of disease on fertility and milk production, and informed their breeding and management decisions, such as when to dispose of a cow. They also made proactive use of tuberculin testing to identify and remove cows affected with bTB, so that they could claim premium prices for ‘TB-free’ milk. They shared their records with the Milk Recording Societies. They, in turn, passed them on to agricultural scientists, who used them to frame and answer wastage survey questions.^41^

It is important to note that many farmers thought quite differently about cattle health and husbandry. Believing that efforts to ‘force’ production were likely to cause disease, they made dairy farming pay by adopting ‘low input, low output’ methods. They were less attentive to the schedule by which cows fell pregnant and more inclined to rely on milk pasteurisation than TB-testing to make milk safe for human consumption.^42^ Nevertheless, the partial version of reality that the wastage surveys represented proved highly influential. It found a receptive audience in the Economic Advisory Council’s Committee on Cattle Diseases, which was appointed in 1932 to address the pressing agricultural and public health question of ‘what practical measures can be taken to secure a reduction of disease among milch cattle.’^43^ The committee’s report claimed that surveys had revealed ‘how serious a part disease plays in the economic management of the *country’s* herds’^44^ and cited ‘unanimity among experienced veterinarians that four diseases are pre-eminently responsible’: brucellosis, bTB, mastitis, and Johne’s disease.^45^ It estimated that the cost of replacing cows lost to wastage amounted to £2.5m per year. Meat condemned as a result of bTB cost another £615 000. The quantity and value of lost milk were impossible to calculate.^46^

These claims perpetuated and extended onto a national scale the disease experiences and priorities of a small minority of dairy farmers. They overlooked the regional nature of the survey data, the unrepresentativeness of its bovine subjects and the atypical outlooks of their owners. By lending impetus to existing public and animal health agendas, they helped to fashion a reality in which the diseases identified were awarded even greater prominence. They provided weight for government decisions to extend bTB control policy and to create a publicly funded Agricultural Research Council (ARC), which adopted animal diseases (particularly brucellosis and to a lesser extent, mastitis) as its highest priority.^47^ As described elsewhere, they also encouraged progressive practising vets, who were seeking additional employment on farms to compensate for the decline in horse usage, to develop and advertise their skills in tackling udder and reproductive diseases.^48^

Wastage survey findings were further reinforced by the results of a calf mortality survey, which the ARC funded in 1936/7. As previously, it relied on data supplied by milk recorded herds. One of the authors was statistician A. Bradford Hill, who is better known today for establishing the first randomised clinical trial (of streptomycin treatment for tuberculosis) and demonstrating the link between smoking and lung cancer.^49^ It concluded, from a geographically distributed survey of 315 dairy herds, that one in seven pregnancies failed to produce an adult cow, and that this was usually due to pre-natal losses caused by abortion or stillbirth.^50^ Hill was the only scientific participant in interwar livestock disease surveying to acknowledge the unrepresentative nature of his ‘highly-selected class’ of subjects, whose health was ‘better than average’.^51^ He was also unique in employing advanced statistical techniques. He used sampling theory to select a geographically representative stratified sample of herds, and applied the chi-square test devised by his former lecturer, Karl Pearson, to reveal that mortality rates showed statistically significant correlations with season and feeding methods (although not with herd size and housing). This approach was ahead of its time. Although adopted by wartime surveyors of other subjects, it did not feature again in animal disease surveys until the 1950s.^52^

In wartime it became a national priority to maximise food production, in particular milk because of its nutritional qualities. The National Veterinary Medical Association (NVMA), the profession’s representative body, attempted to boost the profession’s prospects by further extending the findings of wastage surveys. It appointed a Survey Committee to consider how, in light of the profession’s reservation from active service, vets could be used to the greatest national advantage. The committee was dominated by practising vets while also including those working in science and government. All the members had longstanding interests in udder and reproductive diseases. They did not collect any new disease data, but considered evidence (most likely their own) from vets who had ‘special experience in the subject’. They concluded that brucellosis, infertility, mastitis and Johne’s caused losses of £17m, or 200 million gallons of milk per year. They used these figures as justification for introducing a publicly-funded nationwide ‘scheme for the control of certain diseases of dairy cows’, whose history has been described elsewhere.^53^

The NVMA’s calculations were based on multiple leaps of logic: that the diseases already identified as driving the disposal of cows from milk recorded herds in particular regions were also the most important causes of reduced milk output, nationwide.^54^ They ignored the geographically localised nature of interwar disease surveys, their unusual bovine subjects and the fact that causes of lost productivity did not map neatly onto causes of cattle disposal. For example, husk, or lungworm, was a common problem that caused reduced milk output and prolonged debility, but it did not feature at all in the wastage surveys or in the NVMA’s thinking.^55^ Nevertheless, the government agreed to support NVMA proposals for a ‘survey scheme’, which subsidised practising vets over the period 1942–50 to tackle reproductive and udder diseases in periodic visits to participating farms. Although only 10% of dairy farmers enrolled in the scheme, NVMA leaders celebrated its achievements in improving herd health, raising milk output and advancing veterinary expertise in cattle breeding. They also used it to lay claim to a new identity, as ‘physician of the farm and the guarantor of the nation’s food supply.^56^

The publicity surrounding the survey scheme drew further veterinary, farming and state attention to diseases of the bovine udder and reproductive system and helped to refashion them into threats to the nation’s capacity to defend itself. Elevated by local wastage surveys, extended by national economic costings, targeted by veterinary research and policy, and seized upon by veterinary lobbyists, the causes of premature cattle disposal in a few hundred, unrepresentative and geographically localised herds had come to represent the most significant causes of poor health and productivity in all of the nation’s dairy cows.

## Disease surveying as state veterinary project, 1946–63

After the Second World War, grounds for public concern about dairy cow disease shifted away from the threat it posed to the public’s health, to focus primarily on its implications for agricultural productivity. With food rationing continuing into the 1950s, the British government was keen for farmers to maximise output. It awarded generous subsidies for production under the 1947 Agriculture Act, expanded veterinary and agricultural advisory services and increased research budgets.^57^ The practising veterinary profession was keen to help boost production through further state-supported visits to farms, but NVMA plans for an updated version of the survey scheme did not win farming or government support.^58^ It responded by proposing more disease surveys,^59^ which were intended to improve knowledge of the causes of disease, inform measures for its control and

denote…the real contribution which the profession is making to the increase in provision of foodstuffs for the human population of this country, no less than … the contribution that the profession is making to the economic well-being of the agricultural industry itself.^60^

This proposal, and its intended outcomes, were framed by the NVMA’s Technical Development Committee, which was the successor to its wartime Survey Committee, and similarly dominated by practising vets. It was directly informed by the earlier committee’s wartime mobilisation of survey data and its ambitions for greater professional influence. It also drew on the rising authority and reach of survey techniques. To capture information that could inform policy, the wartime government had conducted surveys on an unprecedented scale, enrolling hundreds of thousands of participants in studies that had significant post-war legacies.^61^ These included the National Farm Survey (1941–3), which made production practices visible to policy makers, informed wartime interventions and post-war planning, and constructed the idea of a ‘national farm’ out of diverse agricultural holdings.^62^ There was also a Wartime Social Survey that conducted over one hundred enquiries in its first three years of operation, many on food and nutrition. It evolved into the Government Social Survey, which became a ‘quintessential research arm of the modern state’^63^ that was ‘implicated in a broader process of building a modern, rational, post-imperial nation’.^64^ It is possible that British vets were inspired by its Survey of Sickness (1943–52). Prompted by the Ministry of Health’s desire for reliable insights into the total amount of illness and its effects, this asked randomly sampled members of the public to report on their health over the preceding three months.^65^

Moving beyond the earlier surveys’ focus on wastage, the NVMA proposed to quantify the range and impact of diseases experienced by dairy cows and to identify the husbandry factors associated with them. Its interest in the latter arose from changes that practising vets were reporting in the patterns of livestock disease that they experienced in their visits to farms. It appeared that heavier stocking densities were facilitating the spread of infectious diseases and that the growing use of ley farming (temporary grassland) was contributing to bloat, grass sickness, mineral deficiency and infertility.^66^ The idea that surveys were capable of demonstrating such links may have been drawn from human medicine, which had been deploying them since the interwar period to try and identify factors contributing to disease in particular communities.^67^ It was given additional impetus by developments in post-war social medicine, which was emphasising the dynamic relationship between health and social factors and seeking to build statistical links between them.^68^

In the forms they created for collecting survey data, members of the Technical Development Committee embedded practitioners’ experiences of disease alongside historical assumptions about the sorts of farmers who were likely to participate and what diseases were important. There was a card for farmers to record instances of disease and disposal, another for husbandry practices and one for the vet to collate this information. Single columns were dedicated to calving events (including abortion), service dates (to indicate infertility) and mastitis, and specific questions were asked in relation to these diseases (e.g., mastitis control measures). Just one column was devoted to all ‘Other conditions (foul, indigestion, retained afterbirth, worms etc)’.^69^ These forms resembled those completed by participants in milk recording schemes, which now covered 17% of cows in England and Wales. Management of milk recording had recently transferred to the Milk Marketing Board (MMB), which had been established in the interwar period as the sole purchaser of milk from British farms. The MMB incorporated data supplied by participating farmers into its new Bureau of Records and assumed the power to use it for research purposes. It also redefined ‘fertility’ by setting 305 out of 365 days as the standard duration of milk recording. This was based on the ‘commercial’ norm (which was not, however, a norm for many farmers) of annual calving.^70^ MMB recording adopted the first of October as the start date of the recording year and so, too, did the NVMA.^71^ The rationalising practices of disease surveying and milk recording therefore continued to reinforce each other, privileging same variety of dairy farming and version of bovine health as the interwar wastage surveys.

The NVMA asked the State Veterinary Service (SVS) to implement its survey.^72^ Officials declined on the grounds of insufficient manpower but promised to reconsider if a pilot was conducted which showed that useful information could be gathered. Consequently, in 1949, the NVMA issued a call via its journal, *Veterinary Record*, for 200 veterinary volunteers, who would each select ten farmers to record all cases of disease in their herds over a one-year period.^73^ Not surprisingly, given the complexity of the survey and expanding veterinary workloads brought on by farming affluence and new antibiotic treatments, only four vets agreed to participate and only three proved suitable.^74^ Nevertheless, in 1950, the SVS stepped in and turned disease surveying into a state veterinary project. The precipitating factor was probably a request made by the ARC for information about the relative economic importance of livestock diseases. The government’s Chief Veterinary Officer, Sir Thomas Dalling, was unable to answer, because

There is no accurate information available on the part which diseases of various kinds play in the agricultural economy of this country. We know that losses from disease, both by deaths of animals and by lowered production must be high, but no survey has ever been made which could form a basis for a near-the-mark estimation. This information is of considerable importance and is recognised in other countries… We are constantly being asked to give views on the economic importance of certain diseases in order to show the value of research and control but have not been able to produce any figures.^75^

The NVMA’s proposals offered a solution to this dilemma. Dalling would have needed little persuasion to accept them because both he and A.W. Stableforth, director of the government’s CVL, had been members of the NVMA’s wartime survey committee. They may even have contributed to its post-war survey planning.^76^ The investigations that they had already launched reveal their weddedness to survey techniques and their attentiveness to the diseases highlighted by earlier surveys. Stableforth was studying mastitis in several hundred randomly selected dairy herds in Surrey, looking at incidence, causes and responses to different penicillin regimes.^77^ Dalling had commissioned a small-scale study of Johne’s disease in Hampshire.^78^ He was also supporting a follow-up to the interwar survey of calf mortality,^79^ which he regarded as ‘one of the most serious causes of loss affecting the agricultural economy of this country’.^80^ This survey made a concerted effort to elucidate the relationship between disease and husbandry, but despite a wealth of data, it made little headway.^81^

Dalling and Stableforth built on and extended the NVMA’s plans into a systematic state-led attempt to determine which diseases afflicted the nation’s livestock and in what quantities, places and species. Their survey programme became one of the defining features of post-war veterinary science. Focussing on ‘the non-notifiable diseases of everyday occurrence, which in the aggregate cause large losses in money and stock’,^82^ it ranged from single diseases to the totality of health experiences, as identified in live animals, dead animals, samples of animals and statistical representations of animals, that were accessed in farms, knackeries, laboratories and production records. The declared goals of the programme were to demonstrate the incidence and economic importance of some of the more important diseases, to indicate the most profitable directions for future enquiry, to gain useful information about and judge the efficacy of control measures and to correlate particular husbandry methods with disease incidence.^83^ Dalling later pointed to an additional, educational purpose, ‘to convince livestock owners of the losses which they themselves are incurring from sickness and deaths among their animals’.^84^ These goals addressed the state’s responsibility under the 1947 Agriculture Act for promoting ‘a healthy and efficient agriculture’.^85^ They recognised the national economic cost of endemic diseases and acknowledged a role for the state in addressing them. Traditionally regarded as private threats to farming profit, these diseases were thereby reframed as public problems that undermined the national economy.

The livestock disease survey programme formed part of a wider state-driven, ‘big science’ project to statistically define the modern nation. Developed in the wake of the war-time National Farm Survey’s construction of a ‘national farm’, in parallel with the Government Social Survey’s account of the human population^86^ and alongside the MMB’s statistical construction of a national dairy herd,^87^ it generated a new concept of the nation’s livestock as a geographical aggregation of animals that were, had been and had the potential to become diseased. The project was bankrolled by the growing budgets of the Ministry of Agriculture and the ARC.^88^ It traversed the CVL, the expanding network of Veterinary Investigation Centres and other research institutions. Surveyors reported their findings in the Annual Reports of the Chief Veterinary Officer, scientific journals and special publications, and at conferences like that described in the opening paragraphs. From 1956, Stableforth hosted annual meetings for scientists engaged in leading survey projects. At least twenty attended, and many more were involved in conducting this work.^89^

Many surveys targeted single diseases, measuring their incidence in particular locations. Sometimes they attempted to delineate associated husbandry practices, and, where multiple microbial agents were implicated, they used laboratory techniques to differentiate between them.^90^ However, most of the available resources were devoted to general studies of the causes of disease, death and wastage, in particular livestock species along the lines proposed by the NVMA. While it had identified what type of data to collect, it had not developed an effective method of collecting it and – reflecting its complete lack of experience in this area – had given no thought to data analysis. To address this problem, Stableforth and Dalling launched a series of regional pilot surveys that ran from 1950 to 1956. Likely informed by a 1950 Medical Research Council conference that explored the potential for operational research in medicine, including its applications to ‘scientific method in field surveys’, they regarded these pilots as a form of operational research that would ‘assess the survey technique and value of data obtained’.^91^

F.W. Withers, who had conducted the 1946–8 survey of calf mortality under Dalling and was unusual in possessing MA and PhD degrees as well as a veterinary qualification, was appointed to lead the regional surveys. From 1952, he worked alongside F.B. Leech (a vet turned statistician) and colleagues at the Rothamsted Experimental Agricultural Station, who brought cutting edge statistical techniques to bear. Rothamsted’s leadership in statistics had been established during R.A. Fisher’s tenure (1919–33) and grew further under F.B. Yates, his successor as Head of Statistics, who had advised the government on the wartime National Farm Survey. In 1954, Yates secured for Rothamsted an Elliott 401, one of the first commercial, programmable electronic computers, which was succeeded by a Ferranti Orion in 1963. Immediately after the war, Yates offered the services of his staff free of charge to researchers funded by the Ministry of Agriculture and ARC. During the 1950s and 1960s, they provided extensive input into agricultural survey projects, particularly those run by the SVS, whose requirements drove them to develop their statistical methods and computing technologies. Although well documented in the Rothamsted annual reports,^92^ this work has gone largely unnoticed by historians of science, whose privileging of Rothamsted’s statistical contributions to experimental methods has obscured the parallel body of work it performed on survey techniques.^93^

The regional surveys replicated the approaches of earlier surveys, in asking farmers to keep records of disease events and hand them over to veterinary recorders who visited at three-monthly intervals. The ARC funded their conduct in Surrey, Berkshire and Wiltshire. Following receipt of US Economic Aid, the programme was expanded in 1953 to Devon, Shropshire, Ayrshire, Lanarkshire and the West Riding of Yorkshire.^94^ In 1957/8, Stableforth was ready to apply survey methods on a national scale. The survey of Disease, Wastage and Husbandry in the British Dairy Herd was billed as a ‘first attempt to make an unbiased assessment on a national basis of the incidence and relative importance of the diseases of dairy cows’.^95^ It aimed to go beyond previous ‘impressionistic or local knowledge…to apply proof to our understanding of the incidence of some dairy farm practices and the constitution of dairy herds in the different regions.’^96^ A follow-up was conducted in 1959/60, followed by a national survey of calf wastage and husbandry in 1962/3. The final section of this article explores the content of these surveys and the assumptions that were embedded in their conduct.

## Rationalisation and resistance

Although billed as a means of deciding which animal diseases were of ‘real importance’ to the nation,^97^ post-war surveys were clearly informed by preconceived ideas about what it meant for a disease to be ‘important’ and which diseases merited this label. Surveyors could have defined ‘importance’ in welfare terms, because the impact of disease on animal well-being did draw comment from farmers and practising vets. However, they did not refer to welfare in their discussions or published reports. Alternatively, reflecting the state-led drive to enhance agricultural output, they could have defined ‘importance’ as lost production. Reduced milk output was a common and costly feature of dairy cow disease and the main reason why (in conjunction with its welfare impacts) farmers were concerned about it. But although post-war surveys spoke frequently about the ‘economic importance’ of disease, they did not make systematic attempts to measure the costs inflicted. The national survey did capture information about the proportion of cows suffering from specified diseases that were culled as a consequence and what prices were received for their carcasses. However, few disease encounters ended this way, meaning that such calculations provided limited insights into the economic impact of livestock disease.^98^

It appears that for the most part, surveyors equated ‘importance’ with disease incidence, which they defined as the percentage of cows affected by a disease in one year. This reading expressed a veterinary view of the world in which the very existence of disease was problematic. Vets tended to see diseases as scientific puzzles in need of veterinary investigation and as farm-level problems requiring veterinary management. The more frequent and puzzling the disease, the more veterinary intervention was required. They assumed that the cost of this intervention would always be less than the losses inflicted by disease; therefore, no particular economic justification was required.^99^ Prior surveys had already shown what were the most frequently occurring diseases. They had organised their research and advisory services around these diseases and saw new surveys as an opportunity to find out what progress had been made.

The SVS’s preconceived notions of disease ‘importance’ are revealed through the forty-nine focussed surveys conducted in the period 1950–60, the vast majority of them by its employees. Thirteen addressed infertility and abortion, nine mastitis, eight Johne’s disease, five brucellosis and two tuberculin testing. The remaining twelve studies (25%) were divided between parasitic diseases, salmonella, white scour, hypomagnesia, neoplasia and bacterial endocarditis.^100^ A similar emphasis can be seen in research and policy. Supported by the Ministry of Agriculture and the ARC, mastitis research was intensifying at various institutions around the country.^101^ In 1956, the CVL created a dedicated ‘Diseases of Breeding’ department. The SVS retained the ‘sterility advisory officers’ it had appointed in wartime, and it continued to roll out subsidised brucellosis vaccines for calves, resulting in a 50% uptake by 1960.^102^

General disease surveys were informed by these developments. Their data recording, analysis and reporting practices privileged the same set of diseases. The first pilot study employed the NVMA’s record cards, thereby perpetuating that organisation’s assumptions about dairy cow health.^103^ Leech then advised their replacement by Cope-Chat punched cards and, subsequently, Hollerith cards. The latter had more space for recording and could be analysed by tabulating machines and eventually electronic computer. On these cards, occurrences of particular diseases were recorded by punching holes in specific locations.^104^ The size of the card did not permit the recording of all of the health events reported by farmers. Consequently, Withers and Leech grouped reports into defined disease categories. The categorisation process was relatively easy for diseases like mastitis and abortion, whose symptoms were well defined and (after a quarter-century of publicity) well known to farmers. Other conditions, for which farmers employed a rich and regionally specific vocabulary that did not align with veterinary terminology (for example, ‘thumps’, ‘fog fever’, ‘hoose’, ‘pankers’ and ‘worm cough’ were all used for the disease known by vets as ‘lungworm’)^105^ were subject to rationalisation by visiting recorders and then bundled into generic, overlapping and somewhat arbitrary groupings. In the national survey, these were listed under three main headings: ‘diseases associated with parturition’ (which gave particular visibility to reproductive problems), ‘infectious diseases’ (which incorporated just seven conditions, of which three were different forms of mastitis and one was Johne’s) and a long list of ‘non-infectious diseases’ (including ‘undiagnosed infertility’). Wastage data was recorded separately and organised into similar categories as those used interwar, enabling comparisons that demonstrated the progress made.^106^

In these ways, surveys continued to reinforce and be reinforced by a simplified version of the diseased bovine body that emphasised its particular susceptibility to diseases of the udder and reproductive system. They also continued to be dominated by the views and experiences of a particular type of farmer, who had a larger herd and was more committed to improving health and productivity than the average. This was in spite of surveyors’ efforts to apply representative sampling (by now an accepted and widely used approach) to the selection of participants.^107^ In their historical study of the wartime National Farm Survey, Murdoch and Ward revealed that statistician Frank Yates deliberately excluded around 70 000 farms that were less than 5 hectares in size and/or where farming did not provide the main source of employment to the occupier. In so doing, he expressed and advanced a view of the farm as a commercial economic proposition rather than a physical unit or way of life.^108^ Leech followed his example. He omitted herds of five cows or less from the national survey and selected 0.5% of herds with 6–20 cows and over 1% of those with more than twenty cows. He argued that this approach made sense, because although there were fewer large herds, they contained more of the nation’s cows. However, it privileged the disease experiences of the average cow over those of the average herd and herd owner. It also obscured regional differences. Certain regions like Devon, where herds were smaller than average, were under-represented, while Cheshire herds, due their larger size, were over-represented. No surveys were conducted in areas of Scotland that Leech defined as ‘physically inaccessible’.^109^ This meant that the ‘national herd’ that disease surveys brought into being was not an aggregation of existing herds but a new entity that awarded certain cows, farmers and regions more importance than others.

The manner in which surveys attempted to rationalise cows and farmers did not go uncontested. As Mold *et al.* have shown in their history of human health surveys after World War Two, subjects could ‘speak back’ by resisting participation, giving incomplete or evasive answers or awarding their own interpretations to questions, thereby threatening the representativeness and accuracy of survey findings.^110^ This was equally true of farmers. Although the SVS surveys attempted to democratise participation by issuing invitations to a random sample of farmers in each of the designated categories, efforts were required to secure participation. In the national survey, farmers were visited by State Veterinary Officers and received encouraging correspondence from the National Farmers Union. Nevertheless, between 10% and 30% of them refused, making it impossible to build collective disease knowledge out of their private experiences. Reportedly, they were reluctant to spend time on an activity that would not benefit them personally or to admit the existence of health problems. Leech did attempt to balance participation by TB ‘designated’ and ‘non-designated’ herds – labels that referred to the progress that farmers were making towards bTB eradication^111^ – but more refusals were received from the latter than the former, meaning that again, progressive farmers were over-represented.^112^

Those farmers who did agree to participate could interpret disease events in quite different ways, and award different labels and varying degrees of importance to them. For example, terms like ‘abortion’, ‘still-birth’ and ‘premature birth’ were applied inconsistently. While reports of death were generally definitive, wastage statistics were determined as much by market prices as the state of a cow’s health. Cows culled on account of ‘infertility’ were not necessarily infertile; rather, their breeding had been delayed to a point at which it was judged uneconomic to keep them. Reportedly, farmers’ record-keeping varied from excellent to primitive. One of the reasons why veterinary recorders visited farmers rather than receiving their reports by post was to elicit further information about poorly recorded disease events. Practising vets assisted this process by adding their own comments to farmers’ record sheets when they visited diseased animals. Some vets believed that all of the recording should have been entrusted to them because of their advanced diagnostic expertise. However, the cost of their attendance would have been prohibitive and would have skewed the data in different ways, towards those diseases that farmers thought merited veterinary intervention.^113^

Bovine bodies could also ‘speak back’ by resisting the disease categories that veterinary surveyors imposed on them. During the post-war years, these bodies were changing dramatically as scientific research, technological advances and government subsidy regimes encouraged farmers to adopt new breeding and husbandry practices. By 1965, Friesians made up 64.2% of the dairy cows in England and Wales compared to 40.6% a decade previously, and the average dairy cow produced 3 545 litres per lactation compared to 2 545 before the war. Machine milking had almost entirely displaced hand milking; cow houses were being replaced by loose housing and separate dairies; silage and temporary pastures were used increasingly for feeding, and intensive calf fattening systems were on the rise.^114^ Together with the widespread use of antibiotics, these practices altered established disease patterns. For example, practising vets noticed that the incidence and complexity of lameness were increasing, while veterinary researchers showed that the causes of mastitis and abortion were changing. Mineral imbalances, which produced a great diversity of symptoms, were becoming more problematic, and there was increasing evidence of respiratory and gastrointestinal disease ‘complexes’ in which multiple microbial agents, including viruses, were implicated.^115^

Increasing attempts were made to capture these problems in single disease surveys. Of the twenty-three reports published between 1962 and 1965, just ten addressed the usual udder and reproductive diseases while thirteen examined other conditions.^116^ However, general disease surveys were unable to accommodate the changing state of bovine health. Because they focussed on disease incidence among regional or national populations, they did not capture the impact on individual herds of serious problems that were not widely distributed. Their reliance on farmer-reported symptoms meant they were incapable of capturing the changing causes and pathologies of disease, while in classifying it as infectious or non-infectious, they diminished the visibility of complex problems for which both were true. For example, in the 1957/8 national survey report, the disease known to practising vets as ‘lameness’ was represented confusingly and simplistically in two mutually exclusive categories: infectious disease (as ‘foul in foot’) and non-infectious disease (where it was broken down into ‘infection other than foul’ and ‘lameness associated with injury’). These categorisations could not begin to express the multiple pathologies and predisposing causes that practising vets were now recognising for lameness. Had all lame cows been categorised together, the incidence would have been 3.88%, second only to mastitis, but they were not, and because this was not an udder or reproductive disease, the authors of the report commented only briefly on it.^117^

The data extracted from bovine bodies also resisted manipulation. It took surveyors so long to process and analyse that by the time they reported, livestock production practices and disease demographics had moved on.^118^ It took two years from the completion of data collection in 1958 to the publication of the first national survey. Most of this time was spent in processing 20 000 Hollerith record cards. Findings from the 1958/9 survey did not appear until 1964. This was in spite of the development of a computerised General Survey Programme by Rothamsted workers, which enabled part of the 1957/8 national survey and the entire 1958/59 survey to be analysed by electronic computer. While computerisation promised to speed up data analysis, weed out field errors in recording and perform more complex calculations,^119^ it was not capable of conducting the sorts of multivariate analyses that could correlate husbandry practices with disease. Such calculations were extremely time consuming to perform by hand. Consequently, in the national surveys, husbandry practices (e.g. milking methods, use of brucellosis vaccine and use of ley pastures) were reported separately from disease, with correlations performed only for herd size, with some additional commentary on season, region and breed.^120^

The challenges that surveyors encountered in general dairy surveys were accentuated in a subsequent survey of calf wastage and husbandry in Britain. Conducted in 1962/3 on 1 657 farm holdings, it probably spelt the death knell for the SVS’s disease survey programme. It had characteristically ambitious aims: to gain an overview of calf rearing methods, to estimate losses, identify their causes at post-mortem and determine associations with different management systems. Only 61% of farmers invited to participate in the survey did so. Because farm recording was restricted to the clinical signs that any farmer could recognise and report correctly, the findings simply reinforced existing veterinary perceptions that respiratory and gastrointestinal diseases were important to calf health. Delays in transporting carcasses to laboratories meant that post-mortem examinations produced few insights. It took five years to transfer data to punched cards, to develop a programme capable of analysing it on Rothamsted’s new Orion computer, to conduct the analysis and publish the findings. Even then, the effects of husbandry on calf health proved impossible to disentangle.^121^ One member of the Veterinary Investigation Service complained, ‘No-one in VIS will be satisfied with this report and many will be alarmed by it’. He pointed out that colleagues had criticised the design of the survey at the outset and that its findings were unusable and likely to be ‘a serious embarrassment’ to the SVS.^122^

Leech and Withers were aware of the ways in which farmers, cows and diseases resisted the rationalising methods of the disease survey, but were either unable or thought it unnecessary to do anything about them. They saw these as methodological difficulties rather problems of principle.^123^ Other surveyors, who met annually at the CVL, adopted a similar focus on the practicalities of who was surveying which diseases and how.^124^ They did not discuss what surveys were discovering and how this information should be used. Evidence suggests that they failed to achieve the objectives laid down earlier by Dalling. Their methods proved incapable of correlating particular diseases with husbandry methods. As the demographics of livestock disease changed, their selective interpretation of it, and their claim to be measuring the incidence and economic importance of some of the *more important* diseases, grew less convincing. A study published in 1966 suggested that the survey programme had failed in its intention to indicate future directions for research. In fact, there was a considerable mismatch between the amount of research that had been performed on particular cattle diseases in the previous five years, relative to their importance as measured by the national survey and assessed by practising vets. While long-recognised problems like infertility were well-investigated, other prevalent conditions like lameness and calf diarrhoea remained in scientific obscurity.^125^

A write-up of the survey programme that appeared in the 1965 official history of state veterinary medicine was brief and downbeat. It claimed that surveys ‘confirm some impressions that have long been held, and correct others. If repeated, the general surveys will show trends in disease’.^126^ However, there was to be no repetition. With Stableforth’s retirement in 1963, surveys lost their champion. Shortly afterwards, the government began to squeeze the Ministry of Agriculture’s budget and to demand more evidence that its veterinary research and advisory services represented good value for money.^127^ The MMB was already publishing cheap, quick (although admittedly unrepresentative) annual analyses of wastage in milk recorded herds,^128^ making Stableforth’s successor, Ivor Field, disinclined to pursue more extended, expensive analyses – although he did continue to support focussed ‘investigatory’ surveys that addressed defined research problems.^129^ Field also drove the development of a quite different way of knowing the health of livestock populations. This was Veterinary Investigation Diagnosis Analysis (VIDA), a national collation of the disease diagnoses made at the twenty-one Veterinary Investigation Centres in England and Wales on clinical specimens submitted by practising vets. It produced a skewed disease picture because conditions on which vets needed no diagnostic assistance were not represented. Nevertheless, once computerised, VIDA (which is still used today) enabled rapid, cheap monitoring of the changing regional and national situation, heralding a permanent shift in emphasis from livestock disease surveying to disease surveillance.^130^

## Conclusion

Despite its ignominious end and the barest of mentions in the official history of the SVS, the disease survey was, in its time, a highly influential and extensively applied method, which dominated British efforts to make the diseases of livestock collectively knowable for around thirty-five years. Focussing particularly on its general applications to dairy cows and calves, this article has traced its evolution from localised studies of wastage to resource-intensive, nationwide analyses of disease and husbandry. It has explained why surveys were promoted successively by agricultural scientists, leaders of the veterinary profession and the government’s SVS as part of a wider movement to survey the nation’s human and non-human resources. Responding to evolving public health and agricultural and veterinary professional agendas, disease surveys enabled the state to extend its gaze beyond a handful of notifiable animal diseases to a host of other ‘everyday’ threats to livestock health. By selectively organising cows and farmers into a national herd and mapping the diseases to which it was subject, they created public knowledge out of farmers’ private encounters with diseased dairy cows.

The shifting fortunes of disease surveys have been interpreted in light of John Law’s observation that surveys prove sustainable when they both create convincing representations of reality and then generate the realities that those representations depict.^131^ I have shown that although surveyors aimed to conduct ‘unbiased’ assessments of dairy cow disease and wastage, they actually created, circulated, extended and enacted a highly selective version of the diseased bovine body, which privileged the diseases that they, and the nation’s progressive farmers, expected and were able to see. This approach had its roots in the interwar period, when diseases of the dairy cow’s udder and reproductive system were problematised by production-oriented farmers, made visible by milk recording practices, targeted by research and control policy and made the focus of veterinary wartime endeavours. Demonstrating the power of surveys to remake the world, it shaped the recommendations of the Economic Advisory Council’s Committee on Cattle Diseases, the NVMA’s Survey Scheme and the pursuit of veterinary research. Together, these initiatives elevated the diseases identified in surveys of wastage in selected, geographically-bounded herds into the most important causes of lost productivity in all of the nation’s dairy cattle. The methods, assumptions and personnel responsible for these developments exerted a continuing influence over the SVS’s post-war survey programme, which then reinforced and perpetuated the same version of the diseased bovine body.

Paradoxically, as the ambition, profile, resourcing and statistical sophistication of disease surveys reached new heights, their findings became less convincing. Contrary to Foucauldian interpretations, which emphasise the power of surveys to statistically represent their subjects and render them governable, in the SVS’s programme, survey subjects resisted, and modes of representing them proved inadequate for their governance. Although the survey programme attempted to democratise farming participation in accordance with statistical sampling theory, surveyors found that farmers ‘spoke back’, and that computing technologies were unable to answer their questions about the relationships between disease and husbandry. Locked into a particular way of viewing, recording and processing information extracted from bovine bodies, survey techniques could not be adapted to new realities when those bodies and their diseases changed. The world as represented by disease surveys diverged increasingly from that experienced by practising vets, farmers and cows. As a consequence, state veterinary aspirations to illuminate the patterns, causes and impacts of dairy cow disease were not fulfilled, and the survey lost its authority as the prime method of collectively knowing the health of the nation’s livestock.

## Footnotes

1

F.W. Withers , ‘Survey of Diseases of Dairy Cattle’, Veterinary Record, 67 (1955), 970–3.

2

G.B.S. Heath , ‘Survey of Sheep Diseases’, Veterinary Record, 67 (1955), 980–3.

3

J.F. Gracey , ‘Survey of Pig Losses’, Veterinary Record, 67 (1955), 984–90.

4 Various, ‘BVA Congress discussion’, *Veterinary Record*, 67 (1955), 990.

5
*Ibid.*, 993.

6
*Ibid.*, 990–5.

7

Volker Hess and J. Andrew Mendelsohn , ‘Case and Series: Medical Knowledge and Paper Technology, 1600-1900’, History of Science, xlviii (2010), 287–314 .

8

Michel Foucault , The Birth of the Clinic: An Archaeology of Medical Perception, Andrew Sheridan (trans.), ([S.I.]: Tavistock Publications, 1973); N. Jewson , ‘The Disappearance of the Sick-Man from Medical Cosmology, 1770–1870’, Sociology, 10, 2 (1976), 225–44.

9

John Eyler , Victorian Social Medicine: The Ideas and Methods of William Farr (London: John Hopkins Press, 1979).

10

Graham Mooney , Intrusive Interventions: Public Health, Domestic Space, and Infectious Disease Surveillance in England, 1840–1914 (Rochester: University of Rochester Press, 2015).

11

Madeleine Mayhew , ‘The 1930s Nutrition Controversy’, Journal of Contemporary History, 23, 3 (1988), 445–64; Daisy Payling , ‘“The People Who Write to Us Are the People Who Don’t Like Us”: Class, Gender, and Citizenship in the Survey of Sickness, 1943–1952’, Journal of British Studies, 59, 2 (2020), 315–42.

12

T.M. Porter , Trust in Numbers: The Pursuit of Objectivity in Science and Public Life (Chichester: Princeton University Press, 1995)10.1177/03063129902900400711623934; Tom Crook , Governing Systems: Modernity and the Making of Public Health in England, 1830–1910 (Oakland: University of California Press, 2016); A. Mold , et al., Placing the Public in Public Health in Post-War Britain, 1948–2012 (Cham, Switzerland: Palgrave Macmillan, 2019)37428850.

13 For a general analysis of animal disease historiography, see A. Woods , ‘Animals and Disease’, in M Jackson (ed.), Routledge History of Disease (London: Routledge, 2017), 147–64. Also see Susan Jones and Peter Koolmees , A Concise History of Veterinary Medicine (Cambridge: Cambridge University Press, 2022). Surveys are mentioned only in passing by the official history of the State Veterinary Service: Anon., *Animal Health, a Centenary, 1865-1965* (London: HMSO, 1965), 363. Their interwar significance in highlighting reproductive problems is discussed in Abigail Woods , ‘The Farm as Clinic: Veterinary Expertise and the Transformation of Dairy Farming, 1930-50’, Studies in History and Philosophy of Biological and Biomedical Sciences, 38 (2007), 462–871754384110.1016/j.shpsc.2007.03.009.

14

D.E. Gray and M.E. Stebbings , ‘An Annotated Bibliography of Surveys of the Incidence of Diseases of Farm Animals and Birds in Great Britain Published During 1931 – 1961’, British Veterinary Journal, 118 (1962), 399–404, 443–6, 494–8 and 534–40; D.E. Gray and M.E. Stebbings , *op. cit.*, British Veterinary Journal, 119 (1963), 42–6 and 83–8; Elizabeth Stebbings and D.E. Gray , ‘An Annotated Bibliography of Surveys of the Incidence of Diseases of Farm Animals and Birds in Great Britain, Supplement for 1962-1965’, British Veterinary Journal, 122 (1966), 348–53, 453–7 and 495–9.

15

F.W. Withers , ‘Surveys of Disease in Dairy Cattle in Great Britain’, Outlook on Agriculture, 1 (1956), 116–22: 116.

16

Ministry of Agriculture, A Century of Agricultural Statistics, Great Britain 1866-1966 (London: HMSO, 1968), 75–7.

17

J.C. Gower , ‘Statistics and Agriculture’, Journal of the Royal Statistical Society, Series A (Statistics in Society), 151, 1 (1988), 179–200 ; Jonathan Murdoch and Neil Ward , ‘Governmentality and Territoriality: The Statistical Manufacture of Britain’s “National Farm”’, Political Geography, 16, 4 (1997), 307–24; B. Short , et al., The National Farm Survey, 1941-43 (Oxford: CABI, 2000); Paul Brassley , et al., ‘Accounting for Agriculture: The Origins of the Farm Management Survey’, Agricultural History Review, 61, 1 (2013), 135–53.

18

Louis Moss , The Government Social Survey: A History ([S.I.]: C.O.I., 1959); S. Koven , ‘The Dangers of Castle Building – Surveying the Social Survey’, in M. Bulmer , K. Bales and K.K. Sklar (eds), The Social Survey in Historical Perspective, 1880-1940 (Cambridge: Cambridge University Press, 1991), 368–76; Mike Savage , Identities and Social Change in Britain since 1940: The Politics of Method (Oxford: Oxford University Press, 2010).

19

David Armstrong , Political Anatomy of the Body: Medical Knowledge in Britain in the Twentieth Century (Cambridge: Cambridge University Press, 1983); Helen Tilley , Africa as a Living Laboratory: Empire, Development, and the Problem of Scientific Knowledge, 1870-1950 (Chicago: University of Chicago Press, 2011); Payling, *op. cit.* (note 14), 315–42.

20 For example, Murdoch and Ward, *op. cit.* (note 17), 307–24; James C. Scott . Seeing Like a State: How Certain Schemes to Improve the Human Condition Have Failed (New Haven: Yale University Press, 1999).

21 For example, Savage, *op. cit.* (note 18).

22

John Law , ‘Seeing Like a Survey’, Cultural Sociology, 3, 2 (2009), 239–56: 240.

23

N Wright , ‘Wastage in Dairy Cows’, Scottish Journal of Agriculture, 16 (1933), 31–40 .

24 For example, on the first appearance of foot and mouth disease in Britain, the Royal Agricultural Society issued written questions to members about their experiences. ‘The Epidemic Among Cattle’, *Journal of the Royal Agricultural Society of England*, 1 (1840), cxii–cvi.

25 Anon., *op. cit.* (note 13); Peter Atkins , ‘Lobbying and resistance with regard to policy on bovine tuberculosis: An inside/outside model of Britain, 1900-1939’, in Flurin Condrau and Michael Worboys (eds.), Tuberculosis Then and Now: Perspectives on the History of an Infectious Disease (Montreal: McGill-Queen’s University Press), 189–212 .

26

J. Burner and D.J. Oddy (eds.), The Origin and Development of Food Policies in Europe (Leicester: Leicester University Press, 1994); Peter Koolmees, ‘Veterinary inspection and food hygiene in the twentieth century’, in D.F. Smith and J. Phillips (eds.), Food, Science, Policy and Regulation in the Twentieth Century. International and Comparative Perspectives (London & New York: Routledge, 2000), 53–68 ; Anne Hardy , ‘Professional Advantage and Public Health: British Veterinarians and State Veterinary Services, 1865–1939’, Twentieth Century British History, 14, 1 (2003), 1–23 1268829510.1093/tcbh/14.1.1; Tatsuya Mitsuda , ‘Trichinosis revisited: Scientific Interventions in the Assessment of Meat and Animals in Imperial Germany’, Food and Foodways, 27, 1–2 (2019), 49–73 .

27 Anon., *op. cit.* (note 13).

28 Tilley, *op. cit.* (note 20).

29 Gower, *op. cit.* (note 17); Koven, *op. cit.* (note 18); Brassley *et al., op. cit.* (note 17).

30

Donald MacKenzie , Statistics in Britain, 1865–1930: The Social Construction of Scientific Knowledge (Edinburgh: Edinburgh University Press, 1981).

31 Relevant research is described in J. Hammond , ‘Agricultural physiology’, Science Progress, 24 (1929–1930), 231–38.

32

R.R. Kay and A.C. McCandlish , ‘Factors Affecting the Yield and Quality of Milk: I. The Age of the Cow’, Journal of Agricultural Science, 19 (1929), 342–72; A. Smith and O. Robison , ‘The Average Ages of Cows and Bulls in Six Breeds of Cattle’, Journal of Agricultural Science, 21 (1931), 136–49.

33

N. Wright , ‘Wastage in Dairy Cows’, Scottish Journal of Agriculture, 16 (1933), 31–40 .

34 Economic Advisory Council, ‘Committee on cattle diseases: Report’, Parliamentary Papers, 1933–34, Cmd. 4591, ix, 427.

35 Alex Bowmer, ‘Dipping, Dosing, Drenching: Managing Unhealthy Beasts on British Farms’ (unpublished PhD thesis: King’s College London, 2019).

36

Robert Olby , ‘Social Imperialism and State Support for Agricultural Research in Edwardian Britain’, Annals of Science, 48 (1991), 509–26; Colin J. Holmes , ‘Science and the Farmer: The Development of the Agricultural Advisory Service in England and Wales, 1900-1939’, The Agricultural History Review, 36, 1 (1988), 77–86 .

37 Mayhew, *op. cit.* (note 11), 445–64; Atkins, *op. cit* (note 25); M. French and J. Phillips , Cheated not poisoned? Food regulation in the United Kingdom, 1875–1938 (Manchester: Manchester University Press, 2000), 158–84; Keir Waddington , ‘To Stamp Out “So Terrible a Malady”: Bovine Tuberculosis and Tuberculin Testing in Britain, 1890–1939’, Medical History, 48, 1 (2004), 29–48 1496864410.1017/s0025727300007043PMC546294; Abigail Woods , ‘Science, Disease and Dairy Production in Britain, c.1927 to 1980’, Agricultural History Review, 62 (2014), 294–314 .

38 ‘Wastage’, Oxford English Dictionary, available at https://www.oed.com/view/Entry/226026?redirectedFrom=wastage#eid accessed 9 May 2023.

39

Major Greenwood , ‘Problems of Industrial Organisation’, Journal of the Royal Statistical Society, 82, 2 (1919), 186–221: 187.

40 Woods, *op. cit.* (note 13) (2007).

41

J. Mackintosh , ‘National Milk Records’, Journal of the Ministry of Agriculture, 49 (1942–3), 247–50; Richie Nimmo , ‘Auditing Nature, Enacting Culture: Rationalisation as Disciplinary Purification in Early Twentieth-Century British Dairy Farming’, Journal of Historical Sociology, 21, 2–3 (2008), 272–302 .

42 French and Phillips, *op. cit.* (note 37); Woods, *op. cit.* (note 13) (2007); Atkins, *op. cit.* (note 24).

43 Economic Advisory Council, *op. cit.* (note 34), 1–7, 109–13.

44 Author’s italics.

45 Economic Advisory Council, *op. cit.* (note 34), 12–13.

46
*Ibid.*, 118–20.

47 Report of the ARC for the Period October 1933–September 1935 (London: HMSO, 1936), 77–85; *op. cit.*, October 1935–September 1937 (London: HMSO, 1938), 279–80; Waddington, *op. cit.* (note 37), 29–48; T. DeJager, ‘Pure Science and Practical Interests: The Origins of the Agricultural Research Council, 1930–1937’, *Minerva*, 31 (1993), 129–50.

48 Woods, *op. cit.* (note 13) (2007), 462–87.

49

Anne Hardy and M. Eileen Magnello , ‘Statistical Methods in Epidemiology: Karl Pearson, Ronald Ross, Major Greenwood and Austin Bradford Hill, 1900-1945’, Sozial-und Präventivmedizin, 47, 2 (2002), 80–91213473710.1007/BF01318387.

50

R. Lovell and A. Bradford Hill , ‘A Study of the Mortality Rates of Calves in 335 Herds in England and Wales (Together with Some Limited Observations for Scotland)’, Journal of Dairy Research, 11, 3 (1940), 225–42.

51
*Ibid.,* 226.

52
*Ibid.*, 225–42; T. Porter , ‘Statistics and Statistical Methods’, in T.M. Porter and D. Ross (eds), The Cambridge History of Science, VII, The Modern Social Sciences (Cambridge: Cambridge University Press, 2003), 238–50.

53 Woods, *op. cit.* (note 13) (2007), 462–87.

54

NVMA, ‘Report on Diseases of Farm Livestock’, Veterinary Record, 53 (1941), 3–14 .

55 Bowmer, *op. cit.* (note 35), 71–101.

56 Woods, *op. cit.* (note 13) (2007), 462–87.

57

Paul Brassley et al., The Real Agricultural Revolution: The Transformation of English Farming, 1939-1985 (Woodbridge: Boydell Press, 2021), 94 .

58 Reports of Meetings, 1947–1950, National Archives (hereafter NA) MAF 35/589.

59 Anon., ‘Collection of Accurate Data on the Incidence of Disease’, *Veterinary Record*, 61 (1949), 614–5.

60 Editorial, ‘Data and Records in Relation to Disease Control’, *Veterinary Record*, 61 (1949), 616.

61 Savage, *op. cit.* (note 18).

62 Murdoch and Ward, *op. cit.* (note 17); Short *et al., op. cit.* (note 17); Brassley *op. cit.* (note 17), 143.

63 Savage, *op. cit.* (note 18), 21.

64
*Ibid.*, vii

65 Patrick Slater, *Survey of Sickness October 1943 – December 1945* (London: Government Social Survey Department, 1946); Payling, *op. cit.* (note 14), 315–42.

66 NVMA, ‘Meeting Report: Ley Farming’, *Veterinary Record*, 60 (1948), 406–10; Thomas Dalling, ‘Relationship of Animal Health to Production’, *Veterinary Record*, 63 (1951), 570–2,

67 Mervyn Susser, ‘Epidemiology in the United States after World War II: The Evolution of Technique’, *Epidemiologic Reviews,* 7 (1985), 147–77.

68 An explicit reference to this agenda was made by made by R. Lovell, author of the interwar calf wastage survey, in his 1949 Presidential Address to the Section of Comparative Medicine: R Lovell , ‘The Recognition of Subsidiary Factors in the Aetiology of Infective Diseases’, Proceedings of the Royal Society of Medicine, 43 (1950), 1–10 1540437910.1177/003591575004300101PMC2081267. On social medicine, see Dorothy Porter , ‘Social Medicine and the New Society: Medicine and Scientific Humanism in Mid-Twentieth Century Britain’, Journal of Historical Sociology, 9 (1996), 168–87.

69 Anon., ‘Collection of Accurate Data’, *Veterinary Record*, 61 (1949), 614–5.

70 Based on a nine-month gestation period, a one-month rest post-calving, and a two-month window – comprising three oestrus cycles – in which the cow would be bred again. Cows would be milked throughout, except for the two months leading up to calving.

71

NVMA, ‘Meeting Report’, Veterinary Record 59 (1947), 627–820270569; MMB, ‘Progress in Milk Recording: The Bureau of Records’, Veterinary Record 60 (1948), 587 ; S. Baker , Milk to Market: Forty Years of Milk Marketing (Heinemann: London, 1973).

72 The SVS had been formally created out of existing government veterinary services in 1937. It consisted of a policy team at head office, a field division, and the Central Veterinary Laboratory with its associated provincial Veterinary Investigation Service. Anon., *op. cit.* (note 13) (1965).

73 Anon., *op. cit.* (note 69), 614.

74 F.W. Withers comment, Survey Meeting, 13 January 1956, NA MAF 189/872.

75 T. Dalling, Draft letter, 23 July 1951, NA MAF 189/463.

76 Anon., ‘Dr Stableforth appointed Director of the Ministry’s Veterinary Laboratory’, *Veterinary Record*, 62 (1950), 195; Anon., ‘Professor Thomas Dalling knighted’, *Veterinary Record*, 63 (1951), 9–10.

77

A.W. Stableforth ‘Field Trials in the Control of Bovine Mastitis’, Veterinary Record 61 (1949), 270 ; A.W. Stableforth , ‘Bovine Mastitis with Particular Regard to Eradication of Streptococcus Agalactiae’, Veterinary Record, 62 (1950), 219–241541329810.1136/vr.62.15.219.

78

K.D. Downham , ‘Johne’s Disease: A Preliminary Note upon the Prevention and Control of the Disease’, British Veterinary Journal, 107, 7 (1951), 309–11. Also see Correspondence and Report, 1948–50, NA MAF 189/629.

79 F.W. Withers, ‘ARC Report on a Survey of Calf Mortality and Disease, 1946-48 inclusive’, NA MAF 189/463.

80 Editorial, ‘Calf mortality’, *Veterinary Journal*, 108 (1952), 311–31: 312.

81 F.W. Withers, *op. cit.* (note 79)

82 F.W. Withers, *op. cit.* (note 15), 116.

83 CVL, ‘Memo to Farmers: Collection of Accurate Data on the Incidence of Disease in Dairy Cattle,’ October 1953, NA MAF 189/463.

84

T. Dalling , ‘Surveys of Livestock Diseases’, British Veterinary Journal, 117, 6 (1961), 256–66: 256.

85 Brassley *et al., op. cit.* (note 57), 94.

86 Savage, *op. cit.* (note 18).

87 MMB, *op. cit.* (note 71), 587.

88 The ARC’s budget increased from £0.3m in 1945–1946 to £5.6m in 1960–1961. Soraya de Chadarevian , Designs for Life: Molecular Biology After World War II (Cambridge: Cambridge University Press, 2002), 36 .

89 Minutes of Survey Meeting held at Veterinary Laboratory, 13 January 1956, NA MAF 189/872.

90 Gray and Stebbings, *op. cit.* (note 14), 494–8, 534–40.

91 Withers, *op. cit.* (note 15), 117; *Medical Research Council, The Application of Scientific Methods to Industrial Service Medicine* (London: HMSO, 1950).

92 The electronic Rothamsted Documents Archive hosts the Annual Reports of the Statistics Department, available at http://www.era.rothamsted.ac.uk/eradoc/books/1 (accessed 9 May 2023).

93

M.J.R. Healy , ‘Frank Yates, 1902-1994: The Work of a Statistician’, International Statistical Review, 63, 3 (1995), 271–88; G. Parolini, ‘Making Sense of Figures: Statistics, Computing and Information Technologies in Agriculture and Biology in Britain, 1920s-60s’ (unpublished PhD thesis: University of Bologna, 2013).

94 Reports and Correspondence, 1950–6, NA MAF 189/463.

95

F.B. Leech , Muriel Davies , W.D. Macrae and F.W. Withers , Disease, Wastage and Husbandry in the British Dairy Herd: Report of a National Survey in 1957-58 (London: HMSO, 1960), 1 .

96
*Ibid.*, v.

97
*Ibid.*, v.

98
*Ibid.*

99

V. Beynon , ‘The Role of the Veterinary Surgeon in British Agriculture’, Veterinary Record, 75 (1963), 1436–48; M. Fussell , ‘The Profession and Agricultural Economics’, Veterinary Record, 83 (1968), 82 .

100 Gray and Stebbings, *op. cit.* (note 14), 494–8, 534–40.

101 Woods, *op. cit.* (note 37), 305–7.

102 Anon., *op. cit.* (note 13) (2007), 231, 246, 320.

103 Withers, Reports on work done this year, 12 December 1950 and 7 January 1952, NA MAF 189/463.

104 Cope-Chat cards were rectangular in shape and had holes around the margins. The centre of the card was left blank for written information. A knitting needle mounted in a handle was used for sorting. Hollerith cards were more efficient and expensive. Data was coded, checked and then recorded by punching the body of the card. Cards displaying holes in similar places were separated out by a sorting machine and counted by a tabulating machine, while a printer produced a hard copy of the results. Minutes of ‘Disease surveys and vital statistics’ meeting, 12 January 1960, NA MAF 189/874; Denis Ward , ‘The Use of Edge-Punched Cards in Statistical Computation’, Journal of the Royal Statistical Society. Series C, 8, 2 (1959), 104–13; Jon Agar , ‘What Difference Did Computers Make?’, Social Studies of Science, 36, 6 (2006), 869–907: 874.

105 Bowmer, *op. cit.* (note 35), 74.

106 Leech *et al., op. cit.* (note 95); F.B. Leech , ‘A Critique of the Methods and Results of the British National Surveys of Disease in Farm Animals. I. Discussion of the Surveys’, British Veterinary Journal, 127, 11 (1971), 511–22516782410.1016/s0007-1935(17)37284-6.

107 Porter, *op. cit.* (note 52). Participants were identified using the MMB’s records bureau; 5% of regional herds were enrolled in pilot studies and 1%, or 1 500 herds, in the national study. Leech *et al., op. cit.* (note 95).

108 Murdoch and Ward, *op. cit.* (note 17), 316–7.

109 Leech *et al., op. cit.* (note 95), 2.

110 Mold *et al., op. cit.* (note 12), 67–97.

111 Anon., *op. cit.* (note 13) (2007), 224–6.

112 Withers, *op. cit.* (notes 1, 15, 95); F.W. Withers , ‘Wastage and Disease Incidence in Dairy Herds’, Veterinary Record, 67 (1955), 605–12.

113
*Ibid.*

114 Brassley *et al.*, *op. cit.* (note 57), 114, 119.

115 S. L Hignett, ‘Farm Health Problems – Where Does Britain Stand Today’, followed by congress discussion, *Veterinary Record*, 68 (1956), 887–900; A. B. Paterson, ‘Virus Diseases in Calves’, *Veterinary Record*, 74 (1962), 1384–94; Ministry of Agriculture, Fisheries and Food, *Diseases of Cattle: Bulletin No 160, 2nd ed.* (London: HMSO, 1964).

116 Stebbings and Gray, *op. cit.* (note 14), 348–53.

117 Leech *et al., op. cit.* (note 95), 27–8; Leech, *op. cit.* (note 106), 515.

118

A. Stewart and L.K. O’Connor , ‘Wastage and Culling in Private Milk Records Herds, 1955-6’, Veterinary Record, 69 (1957), 1021–5, 1021; Leech, *op. cit.* (note 106), 512.

119 Anon., ‘The Introduction of Electronic Computers in Research Statistics’, in *Report of the Agricultural Research Council for the Year 1957-58* (London: HMSO, 1959), 62–8.

120 Leech *et al., op. cit.* (note 95).

121 Rothamsted Experimental Station, ‘Rothamsted Report for 1966’, available at http://www.era.rothamsted.ac.uk/eradoc/books/1 (accessed 9 May 2023); F.B. Leech , W.D. Macrae and D.W. Menzies , Calf Wastage and Husbandry in Britain 1962-63 (London: HMSO, 1968); Leech, *op. cit.* (note 106), 517–21.

122 W.H. Parker, letter to D.W. Menzies, 31 January 1966, NA MAF 364/27.

123 Withers, *op. cit* (note 1); Leech, *op. cit* (note 106).

124 Minutes of ‘Disease Surveys and Vital Statistics’ meetings, 1956–64, NA MAF 189/872 – 189/877.

125

J.M. Payne , ‘The Importance of Cattle Diseases in the United Kingdom in Relation to the Research Carried Out Upon Them’, British Veterinary Journal, 122 (1966), 183–9.

126 Anon., *op. cit.* (note 13) (2007), 363.

127 Minutes of ‘MAFF Animal Health and Advisory Services’ meeting, 1 December 1965. NA MAF 287/353.

128 Stewart and O’Connor, *op. cit.* (note 118), 1021–5.

129 Leech distinguished this type of ‘investigatory’ survey from the more general ‘economic’ surveys that aimed to discover what diseases were ‘out there.’ This appears to have been a retrospective definition. F.B. Leech , ‘A Critique of the Methods and Results of the British National Surveys of Disease in Farm Animals. II. Some General Remarks on Population Surveys of Farm Animal Disease’, British Veterinary Journal, 127, 12 (1971), 587–92516931210.1016/s0007-1935(17)37236-6.

130

M.E. Hugh-Jones , D.W. Ivory , R.M. Loosmore and J. Gibbins , ‘Veterinary Investigation Diagnosis Analysis’, Veterinary Record, 84 (1969), 304–7581310810.1136/vr.84.12.304; Animal Health and Plant Agency, ‘VIDA’ (2022), available at http://apha.defra.gov.uk/vet-gateway/surveillance/scanning/vida.htm (accessed 9 May 2023).

131 Law, *op. cit.* (note 22), 240.

